# Impact of coenzyme Q10 as an adjuvant therapy to letrozole on spermiogram results and sex hormone levels in Iraqi men with infertility; randomized open label comparative study

**DOI:** 10.12688/f1000research.131985.1

**Published:** 2023-09-01

**Authors:** Essa Bahauldeen Fadhil, Mohammed Mahmood Mohammed ‎, Ula M. Alkawaz

**Affiliations:** 1 Department of Clinical Pharmacy, College of Pharmacy, Mustansiriyah University, Baghdad, Baghdad Governorate, Iraq; 2 High Institute of Infertility Diagnosis and Assisted Reproductive Technologies, Al-Nahrain University, Baghdad, Baghdad Governorate, Iraq

**Keywords:** Male, infertility, spermatogenesis, idiopathic

## Abstract

**Background:** Worldwide, infertility affects about 15% of reproductive-age couples. In many cases, infertility can't be treated, however new treatment options with promising value have been involved in recent clinical trials. The aim of this clinical trial was to evaluate the impacts of adding coenzyme Q10 (CoQ10) to letrozole on the results of spermiogram and sex hormone tests in men diagnosed with idiopathic oligoasthenoteratozoospermia (iOAT) syndrome, which is a type of male defective spermatogenesis of unknown etiology.

**Methods:** This randomized, open-label, parallel two-arm interventional study included 67 adult male patients aged 18-60 years with a confirmed diagnosis of iOAT syndrome recruited from The High Institute for Infertility Diagnosis & Assisted Reproduction Technologies/Nahrain University. Patients were randomly separated into two groups, Group A included 29 patients treated with letrozole 2.5 mg tablet orally twice a week, Group B included 38 patients treated with a combination of letrozole 2.5 mg tablet orally twice a week plus CoQ10 400 mg per day. Both groups completed treatment for three months. Semen samples, serum follicle-stimulating hormone (FSH), estradiol (E _2_), and testosterone (T) were analyzed at day one, and at the end of month one, two and three.

**Results:** Both groups showed that sperm concentration, normal morphology, total sperm count and motility, serum testosterone and FSH levels, and T/E _2_ ratio were significantly increased, while estradiol levels significantly decreased after three months of treatment. Seminal fluid volume changed significantly in group A only. In comparing between the two groups, all measured parameters, apart from sperm motility and FSH level, demonstrated a significant difference after three months of treatment, while sperm volume reached significant value after only two months of therapy.

**Conclusions:** CoQ10 as adjuvant treatment to letrozole effectively improved most of the tested sperm parameters in Iraqi men with iOAT.

**Registration:** ClinicalTrials.gov ( NCT05847257, May 6, 2023).

## Introduction

The World Health Organization (WHO) defines infertility as “the incapability of a couple to conceive in spite of having regular sexual activity for at least one year without using any contraceptive methods”. ^1^ Around the world, approximately 15% of reproductive-age couples are struggling with infertility, ^2^ about half of these issues are related to male factors. ^3^ There are many causes contributing to male infertility, including infection, ^4^ alteration in organ function, ^5^ environmental factors, genetic factors, ^6^ and sex hormone disturbance. ^7^

Idiopathic oligoasthenoteratozoospermia (iOAT) is associated with faulty spermatogenesis and is characterized by unusually low sperm count, motility, and a large number of dysmorphic spermatozoa in the ejaculate, The etiology of which is unclear and is frequently considered undetectable by standard laboratory techniques. About 30% of OAT patients are identified as idiopathic, and idiopathic testicular abnormalities cause the most severe cases of OAT. ^8^ Although established disorders such as varicocele, cryptorchidism, and hypogonadism are identifiable causes of OAT and infertility, approximately 25% of these individuals do not have a known explanation behind their irregular semen analysis. ^9^^,^^10^

A complex interaction of hormones that act centrally and intratesticular is necessary for spermatogenesis. In response to the activity of gonadotropin-releasing hormone (GnRH) from the hypothalamus, the anterior pituitary secretes luteinizing hormone (LH) and follicle-stimulating hormone (FSH). In the testis, FSH operates on Sertoli cells to stimulate the maturation of spermatogonia. LH has an influence on Leydig cells promoting testosterone synthesis. Local testosterone concentrations must be significantly greater than serum levels for effective spermatogenesis. Then, by its effects on Sertoli cells, this intratesticular testosterone indirectly promotes the development of germ cells. ^7^

Many hormones are participating in the regulation of spermatogenesis. LH stimulates Leydig cells to release testosterone to promote the production of sperm and virilization and exerts negative feedback to suppress the release of LH and FSH from the pituitary gland. FSH promotes Sertoli cells to support spermatogenesis to release inhibin B that has negative feedback effects to inhibit FSH secretion. FSH is required to establish spermatogenesis and it’s very important to understand that testes will produce lower numbers and poorer quality of sperm with only FSH stimulation, while LH is necessary to obtain both quantity and quality of sperm production, as a result neither FSH alone nor LH alone is sufficient to produce high quality sperm. ^11^

Aromatase is a cytochrome p-450 enzyme present in the adipose tissue, testes, liver, and female reproductive organs that has roles in converting testosterone (T) to estradiol (E _2_) and androstenedione to estrone. Aromatase inhibitors block the conversion of T to E _2_; therefore, the level of testosterone is increased while the estrogen level is suppressed. As the endogenous testosterone levels rise, and in combination with the reduction in the estrogen suppressing role on the hypothalamic–pituitary–gonadal (HPG) axis, spermatogenesis is further stimulated. ^12^ In men, the modification of plasma E _2_ levels to the normal physiological range results in a positive impact on FSH, LH and testosterone levels mediated by an effect on the pituitary gland derived by this reduction. ^13^ Therefore in men with low testosterone levels, aromatase inhibitors enhance testosterone secretion. ^14^

Letrozole belongs to third-generation aromatase inhibitors that inhibit estrogen biosynthesis reversibly and act as an anti-cancer agent for advanced breast cancer. ^15^ The medication dosage form is tablet 2.5 mg and when taken orally it is absorbed readily (bioavailability 99.9%); food has no effects on its absorption. Letrozole is rapidly distributed, its excreted mainly through the urine (about 90%) and its half-life is about two days. ^16^

Oxidative stress (OS) and reactive oxygen species (ROS) are believed to damage the spermatozoa and account for 30 to 80% of infertility cases in men. ^17^ ROS in semen originates from different endogenous and exogenous sources, the endogenous sources include round cells, epithelial cells and leukocytes, while lifestyle factors including drinking alcohol, smoking and ecological factors (such as radiation and toxins) are considered sources of exogenous ROS. ^18^ Increased ROS production causes oxidative stress and reduces the antioxidant capacity of spermatozoa. ^19^ Spermatozoa have plasma membranes that are made up of lipids and polyunsaturated fatty acids, increased levels of ROS make the membrane vulnerable to lipid peroxidation and damage. ^20^ The lower motility and reduced fluidity of membranes in sperm occur due to lipid peroxidation and are associated with reduced ability of sperm to fertilize. ^21^ Coenzyme Q10 (CoQ10) is a fat soluble vitamin-like molecule naturally found in cell membranes in the human body and it is naturally found in our diet and can be synthesized endogenously. ^22^ CoQ10 has antioxidant effects and is involved in the production of mitochondrial energy, which is important for maintaining the power source of spermatozoa and provide protection to their membranes from damage through lipid peroxidation. Therefore, it’s considered among the most extensively utilized antioxidants as an option to treat idiopathic infertility in men. ^23^ The aim of the current clinical trial was to evaluate the impacts of adding CoQ10 to letrozole on spermiogram results and sex hormone levels in men diagnosed with iOAT syndrome.

## Methods

### Ethical statement

Written informed consent was obtained from each participant for participation and publication of clinical information. The study was approved by both the institutional regulation board of the Department of Clinical Pharmacy/College of Pharmacy/Mustansiriyah University (ID number: 3983, date: 14 ^th^ December 2021), and the High Institute for Infertility Diagnosis & Assisted Reproductive Technologies/Nahrain University (Date: 3 ^rd^ November 2021; Ethical code: A21024). ^24^ The recruitment period started on the 1 ^st^ of January 2022 after both faculties approved the study. The study followed the Declaration of Helsinki (2008) for research on human subjects and its later amendments. This trial was registered with ClinicalTrials.gov ( NCT05847257) on May 6, 2023. Due to the recent introduction of required clinical trials registration in Iraq, we registered the study retrospectively to assure its transparency. This study adhered to the CONSORT guidelines, ^38^ no harms or unintended effects have been reported for this study.

### Trial design

**Figure 1.**
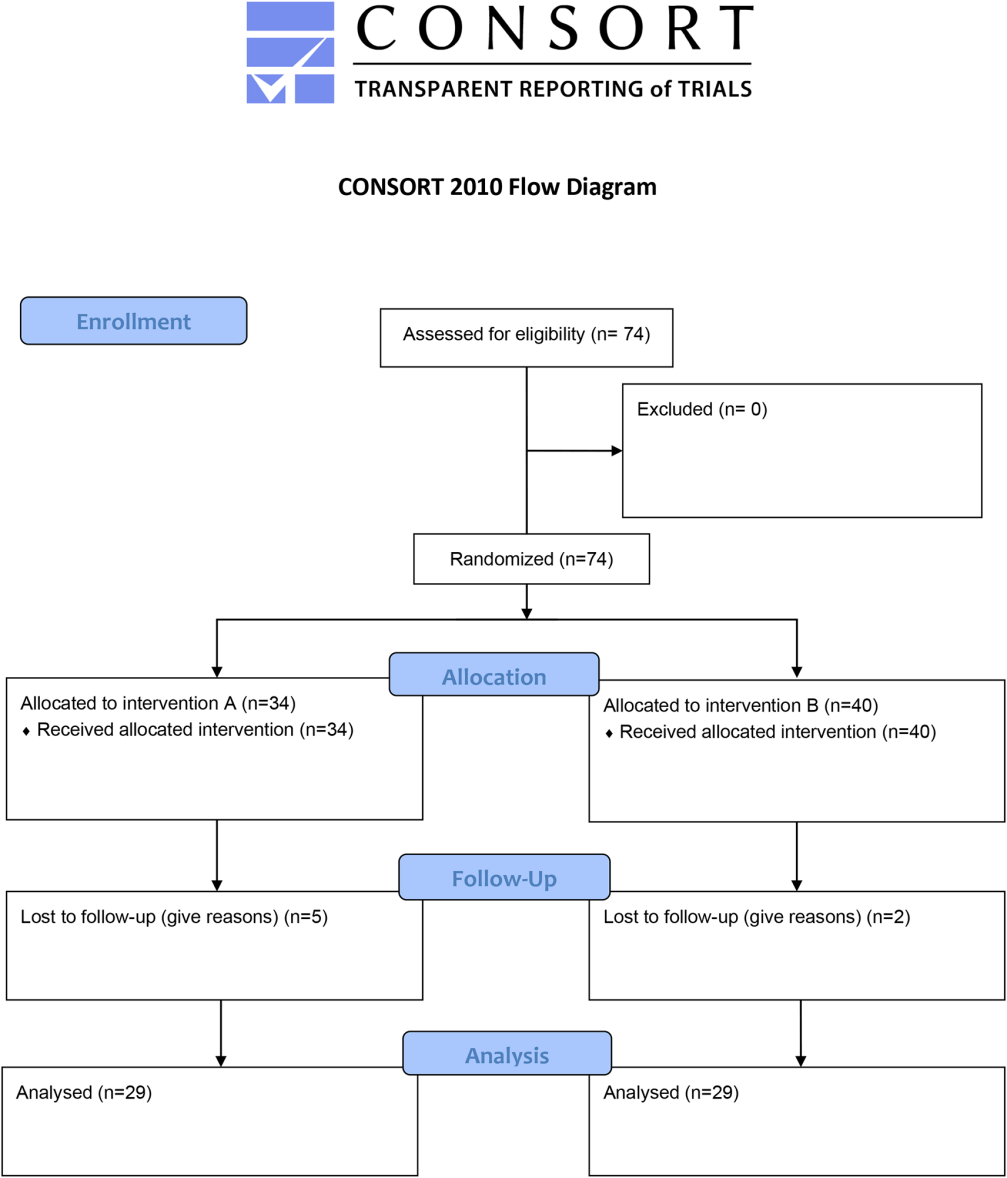
Flow chart of the trial.

This study was a randomized, open-label, parallel two-arm interventional study. The study initially included 74 patients, on follow-up seven cases were lost and the final analysis involved 67 cases. These patients were further divided into two groups randomly, group A (29) patients received letrozole 2.5 mg (Letrozole® 2.5 mg, Accord Healthcare Limited, UK) twice weekly for three months, and group B (38) patients received letrozole 2.5 mg twice a week plus CoQ10 400 mg (CoQ-10®, Natrol, USA) once per day (200 mg twice daily) for three months as illustrated in Figure 1.

### Study settings

Patients with iOAT syndrome were selected during their visit to The High Institute for Infertility Diagnosis & Assisted Reproduction Technologies/Nahrain University. Before taking part, each subject provided their written, informed consent. The duration of this study was from the 1 ^st^ of December 2021 until 1 ^st^ of October 2022.

### Participants

*Inclusion criteria*

1. Adult male patients.
2. Aged 18–60 years.
3. Confirmed diagnosis of iOAT syndrome.

*Exclusion criteria*

1. Patients who have been found to have additional infertility causes, such as varicocele or obstruction of the ejaculatory duct.
2. Those who have had surgery for male factor infertility.
3. Patients with infections such as sexually transmitted infections (STIs).
4. Patients with renal or liver disease.
5. Incomplete patient data.

### Variable measurement

The seminal fluid analysis (SFA) and hormone profile (FSH [VIDAS®FSH, bioMerieux SA, France Cat no.:30 407-01], estradiol [VIDAS®E2II, bioMerieux SA, France Cat no.: 30 431], and testosterone [VIDAS® Testosterone II, bioMerieux SA, France Cat no.:414320]) were assessed at the beginning of the study (baseline value) and at the end of months one, two and three for three consecutive months after taking the medication to measure the possible changes in the studied parameters. The measurement of FSH, LH, and testosterone was performed using enzyme linked florescent assays (ELFAs) (mini VIDAS, bioMerieux SA, France).

### Randomization

The randomization process was done utilizing the online software Research Randomizer. First, the patients were sequentially numbered during the interview and then randomly assigned to one of two groups using the online software.

### Outcomes

*Primary outcomes*

Seminal fluid analysis

Assessment of sperm volume/ ml, sperm concentration/ ml, total sperm count/ml, progressive sperm motility (%), non-progressive sperm motility (%), immotile sperm (%), normal sperm morphology (%). Time Frame: changes in value from baseline to the end of the first, second or third month for three consecutive months of treatment.

*Secondary outcomes*

Hormone profile

Assessment of concentration of serum FSH, concentration of serum estradiol, and concentration of serum testosterone. Time Frame: change in value from baseline to the end of the first, second and third month for three consecutive months of treatment.

### Sample size

A previous study by Peivandi *et al.*, ^25^ showed that letrozole 2.5 mg increases sperm concentrations over three months and demonstrated an in improvement by 33% for sperm parameters (concentration after three months of therapy) so we assumed that the use of CoQ10 will increase this value to 55%. Using MedCalc (RRID:SCR_015044) software version 14.8.1, we arrived at 64 patients for both groups to confirm the null hypothesis (with type I error 5% and type II error of 10%), we increased the sample to 74 to account for maximum possible loss in patients being around 15% without affecting the accuracy of the results.

### Statistical analysis

All analyses were carried using IBM SPSS Statistics (RRID:SCR_016479) version 28.1 (Chicago, USA), repeated measures ANOVA was used to assess the differences in each group across four time periods with post hoc Tukey’s test to assess the pair wise comparisons. An independent t-test was used to assess the difference between each treatment group. A chi-squared test was used to assess the difference in categorical variables. The level of significance was 0.05 (alpha level), and all p-values were two tailed. For sample size calculations MedCalc (RRID:SCR_015044) software version 14.8.1 was used.

## Results

The demographic data and baseline characteristics of all participants are summarized in Table 1. ^38^ In this table, there were non-significant variations among all parameters between the two groups.

**Table 1.** Demographic data and disease characteristics.

Parameters		Group A	Group B	P Value
**Age (years)**		32.6 ± 7.5	35.1 ± 8.3	0.211
**BMI (kg/m** ^**2**^ **)**		31.4 ± 5.1	32.7 ± 5.6	0.313
**Smoking**	**Smoker**	17 (58.6%)	23 (60.5%)	0.875
	**Non-smoker**	12 (41.4%)	15 (39.5%)	
**Duration of infertility (years)**		4.9 ± 2.8	5.4 ± 3.1	0.492

N: Number of patients, % Percentage. BMI, body mass index.

Table 2 shows the results of letrozole effects alone and with CoQ10 on sperm production after one, two and three months. The effects on both groups showed a significant growth in the mean value of sperm concentration and total sperm count in respect to the baseline (p<0.01). However, significant (p<0.05) and non-significant (p>0.05) changes in seminal fluid volume in group A and B were reported, respectively. At the end of the current study, the results revealed significant (P˂0.05) differences between the two groups for all parameters (sperm concentration and total sperm counts) except seminal fluid volume, which was significant only after two months (P>0.05).

**Table 2.** Effect of letrozole alone and in combination with CoQ10 on sperm concentration.

Sperm production		Group A	Group B	P value ^A^
		Mean ± SD	Mean ± SD	
**Volume/ml (ml)**	Baseline	3.1 ± 1 ^a^	3.1 ± 0.9 ^a^	0.975 ^NS^
	First month	2.6 ± 0.9 ^b^	3 ± 0.8 ^a^	0.058 ^NS^
	Second month	2.7 ± 0.7 ^ab^	3.1 ± 1.1 ^a^	0.048 ^*^
	Third month	3 ± 1 ^ab^	3.1 ± 1 ^a^	0.722 ^NS^
**P value** ^**B**^		0.044 ^*^	0.769 ^NS^	
**Sperm concentration/ml (Mil./ml)**	Baseline	8.9 ± 7.9 ^a^	7.3 ± 5.2 ^a^	0.305 ^NS^
	First month	14.1 ± 11.9 ^b^	11.9 ± 9.2) ^b^	0.369 ^NS^
	Second month	17.2 ± 14.1 ^c^	20 ± 14.1 ^c^	0.392 ^NS^
	Third month	16.5 ± 11.9 ^d^	25.2 ± 16.5 ^d^	0.020 ^*^
**P value** ^**B**^		< 0.001 ^**^	< 0.001 ^**^	
**Total sperm count/ml (Million)**	Baseline	26.8 ± 22.4 ^a^	24.8 ± 20.6 ^a^	0.689 ^NS^
	First month	35 ± 25.8 ^ab^	36.5 ± 32 ^b^	0.818 ^NS^
	Second month	46.3 ± 39.7 ^c^	66 ± 60.4 ^c^	0.392 ^NS^
	Third month	52.9 ± 48.8 ^ad^	80.1 ± 62.6 ^cd^	0.029 ^*^
**P value** ^**B**^		0.005 ^**^	< 0.001 ^**^	

Data presented as mean ± SD.

^A^ t-test used to test statistical differences between 2 groups (Horizontally).

^B^ One way repeated measures ANOVA used for comparison between (Time-wise values) within each group. Different small case letters indicate significant difference. NS: No significant changes (p≥0.05).

* significant changes (p<0.05).

** highly significant changes (p<0.01). CoQ10, coenzyme Q10.

Table 3 illustrates the effects of letrozole alone and with CoQ10 on sperm motility and morphology after one, two and three months. Statistically, significant improvement was reported in both groups regarding sperm motility and morphology after three months of treatment. However, comparison between the groups, showed no significant differences (P>0.05) in all parameters except the only sperm morphology was significantly improved in group B compared to that of group A (p<0.05).

**Table 3.** Effect of letrozole alone and in combination with CoQ10 on sperm motility and morphology.

Parameters		Group A	Group B	P value ^A^
		Mean ± SD	Mean ± SD	
**Progressive motility (%)**	Baseline	16.2 ± 16.4 ^a^	11.8 ± 13.2 ^a^	**0.198** ^**NS**^
	First month	26.5 ± 19.8 ^b^	18.8 ± 14.2 ^b^	**0.054** ^**NS**^
	Second month	29 ± 18.4 ^bc^	27.3 ± 16.4 ^c^	**0.673** ^**NS**^
	Third month	27.4 ± 16.3 ^dc^	30.7 ± 15.7 ^dc^	**0.405** ^**NS**^
**P value** ^**B**^		< 0.001 ^**^	< 0.001 ^**^	
**Non-progressive motility (%)**	Baseline	12 ± 9.4 ^a^	12.4 ± 10.6 ^a^	**0.888** ^**NS**^
	First month	12.3 ± 8.4 ^ab^	14.5 ± 11.2 ^ab^	**0.328** ^**NS**^
	Second month	13.2 ± 7.7 ^abc^	14.4 ± 10.1 ^abc^	**0.583** ^**NS**^
	Third month	15.5 ± 8 ^dc^	16 ± 10.2 ^bd^	**0.825** ^**NS**^
**P value** ^**B**^		0.040 ^*^	0.013 ^*^	
**Immotile (%)**	Baseline	66.4 ± 24.6 ^a^	73.4 ± 19.7 ^a^	**0.172** ^**NS**^
	First month	62.7 ± 21 ^ab^	66.4 ± 18.3 ^b^	**0.408** ^**NS**^
	Second month	57.9 ± 20.6 ^ac^	58.5 ± 18.8 ^c^	**0.900** ^**NS**^
	Third month	57.7 ± 17.7 ^ad^	51.3 ± 18.1 ^d^	**0.155** ^**NS**^
**P value** ^**B**^		< 0.001 ^**^	< 0.001 ^**^	
**Normal morphology (%)**	Baseline	1.6 ± 2.28 ^a^	2.1 ± 1.79 ^a^	**0.363** ^**NS**^
	First month	2.5 ± 2.27 ^b^	2.6 ± 2.26 ^ab^	**0.940** ^**NS**^
	Second month	3.4 ± 2.54 ^**c**^	4.4 ± 4.47 ^c^	**0.252** ^**NS**^
	Third month	3.7 ± 2.51 ^d^	5.5 ± 5.13 ^d^	**0.040** ^*^
**P value** ^**B**^		< 0.001 ^**^	< 0.001 ^**^	

Data presented as mean ± SD.

^A^ t-test used to test statistical differences between 2 groups (Horizontally).

^B^ One way repeated measures ANOVA used for comparison between (Time-wise values) within each group. Different small case letters indicate significant difference. NS: No significant changes (p≥0.05).

* significant changes (p<0.05).

** highly significant changes (p<0.01). CoQ10, coenzyme Q10.

Results of comparing the effect of letrozole alone and in combination with CoQ10 on sex hormones after one, two and three months are demonstrated in Table 4. In both groups, a highly significant (p<0.01) increase in testosterone and FSH levels was noticed. Conversely, estradiol levels were significantly decreased (p<0.01). Between the two groups, significant (p<0.05) and highly significant (p<0.001) changes in testosterone and estradiol levels were noticed, respectively, with no significant (p>0.05) changes for FSH levels. Regarding the ratio of T/E _2_, the growth was about 190% and 312% for group A and B from the base line, respectively, the statistical significancy was achieved between the two groups (p<0.05).

**Table 4.** Effect of letrozole alone and in combination with CoQ10 on sex hormones.

Parameters		Group A	Group B	P value ^A^
		Mean ± SD	Mean ± SD	
**Testosterone ng/ml**	Baseline	4 **±** 1.5 ^a^	3.3 **±** 1.3 ^a^	0.059 ^NS^
	First month	5.1 **±** 1.3 ^b^	4.8 **±** 1.2 ^b^	0.370 ^NS^
	Second month	5.8 **±** 1.1 ^b c^	6.2 **±** 1.6 ^c^	0.179 ^NS^
	Third month	6.8 **±** 0.9 ^b c d^	7 **±** 2 ^d^	0.032 ^*^
**P value** ^**B**^		< 0.001 ^**^	< 0.001 ^**^	
**Estradiol Pg/ml**	Baseline	51.8 **±** 10.2 ^a^	50.4 **±** 11.1 ^a^	0.585 ^NS^
	First month	43 **±** 9.5 ^b^	40.8 **±** 8 ^b^	0.274 ^NS^
	Second month	35.6 **±** 8.6 ^c^	33 **±** 7.7 ^c^	0.176 ^NS^
	Third month	31.4 **±** 8.4 ^d^	26.1 **±** 6.6 ^d^	0.005 ^*^
**P value** ^**B**^		< 0.001 ^**^	< 0.001 ^**^	
**FSH mIU/ml**	Baseline	5.5 **±** 1.8 ^a^	6.4 **±** 2.6 ^a^	0.098 ^NS^
	First month	7.1 **±** 2.1 ^b^	8.2 **±** 2.9 ^b^	0.073 ^NS^
	Second month	9.3 **±** 3.5 ^c^	10 **±** 3.6 ^c^	0.402 ^NS^
	Third month	10.5 **±** 3.5 ^d^	11.2 **±** 3.5 ^d^	0.436 ^NS^
**P value** ^**B**^		< 0.001 ^**^	< 0.001 ^**^	
**Ratio T/E**_**2**_	Baseline	8.1 **±** 4.1 ^a^	7 **±** 3.2 ^a^	0.177 ^NS^
	First month	12.9 **±** 6 ^b^	12.3 **±** 3.7 ^b^	0.584 ^NS^
	Second month	17.8 **±** 7.4 ^c^	20 **±** 7.6 ^c^	0.203 ^NS^
	Third month	23.5 **±** 8.1 ^d^	28.9 **±** 11.3 ^d^	0.032 ^*^
**P value** ^**B**^		< 0.001 ^**^	< 0.001 ^**^	

Data presented as mean ± SD.

^A^ t-test used to test statistical differences between 2 groups (Horizontally).

^B^ One way repeated measures ANOVA used for comparison between (Time-wise values) within each group. Different small case letters indicate significant difference. NS: No significant changes (p≥0.05).

* significant changes (p<0.05).

** highly significant changes (p<0.01). CoQ10, coenzyme Q10.

## Discussion

The male reproductive capacity is affected by many demographic factors, with age seemingly the most sensitive factor since aging is negatively correlated with spermatogenesis. ^26^ In the current study, most participants were in their thirties; consequently, a correlation may be present relating to the quantity and quality of sperm. The second important factor is smoking, a study by Jin-Bo Dai *et al*., (2015) confirmed that tobacco smoking is considered a risk factor for developing infertility, smoking cigarettes, water pipes or vapes may contain many harmful chemicals that disrupt the antioxidant status of testes and consequently lead to the interruption of spermatogenesis. ^27^ However, in spite of the adverse consequences of smoking on male fertility, many men are still fertile but they are at risk for becoming infertile. ^28^ More than half of the patients in the current study were smokers and this may have a great influence on the quality of sperms.

In this study, most of the patients were overweight and obese, this may have an association with low quality of spermatozoa. Belloc *et al*., (2014) showed a correlation between high BMI and low semen quality, whereas sperm morphology is not affected. ^29^ In previous clinical trials, letrozole was used alone at a dose of 2.5 mg per day to treat infertility in men, ^30^^,^^31^ while another previous study used a weekly dose of 2.5 mg letrozole to improve testosterone. ^32^ However, the dose of letrozole used in the current study was 2.5 mg twice a week for all patients, this came from the experience of consultants in an attempt to decrease the expected side effects, including loss of libido, which was reported in earlier studies. ^31^^,^^33^

A study by Kooshesh *et al.*, (2020) concluded that the use of letrozole in the treatment of men with iOAT and T: E2 ratio ≤ 10, can successfully increase sperm quality and chromatin integrity, and consequently increase spontaneous pregnancy. ^12^ To a large extent, these finding are in agreement with the current results.

In men, estrogen is mainly produced by the conversion of testosterone to E _2_ *via* aromatase enzymes, ^34^ an excess of estrogens can block the HPG axis, therefore, infertility occurs due to reduced release of FSH and LH. Administration of letrozole can lead to increased testosterone and decreased estradiol production. So by improving the T/E _2_ ratio, this can have positive effects on spermatogenesis and obtaining high quality and quantity of sperm. ^7^

The addition of CoQ10 to letrozole showed notable improvement in the ratio of T/E _2_ (312%) compared to that of using letrozole alone (190%) after three months of treatment. Peivandi *et al.*, (2019) showed a correlation between the improvement in spermatogenesis to the increase in the ratio of T/E _2._^25^ Appasamy *et al*., (2007) suggested that the increased ROS levels may have potential to disrupt hormonal balance and reduce the levels of male sex hormones, therefore resulting in infertility. ^35^ However, administration of CoQ10, which has notable antioxidant properties to scavenge ROS and reverse oxidative stress, results in the improvement of spermiogram parameters. ^36^

Safarinejad (2009) illustrated the effects of CoQ10 on sperm parameters, using 300 mg CoQ10 a day for six months to show enhancement in sperm count, motility and morphology, while decreasing the levels of FSH. ^37^ In the present study, a combination of CoQ10 and letrozole were used for three months, the results indicated improvement in sperm motility, morphology and concentration. Furthermore, assessment of sex hormone levels showed an increase in FSH and testosterone and a decrease in E _2_ levels.

The combined administration of letrozole and CoQ10 led to significant improvements of all spermiogram parameters, this combination appears to have a synergistic effect to obtain higher quality spermatogenesis.

### Study limitations

A limitation of this study includes the small sample size for both groups due to missing many patients during follow-up periods and exclusion criteria mentioned above. In addition, there was a lack of control group due to the difficulty in convincing men who have not been diagnosed with infertility to undertake SFA and hormone analysis. Thirdly, no sensitivity analyses were conducted for the confounding variables. Finally, ROS in seminal fluid was not measured due to the lack of availability of tools to measure ROS in semen.

## Conclusions

The combined use of letrozole and CoQ10 was found to improve sperm parameters and patients showed a boosted improvement in all spermiogram parameters compared to that of patients administered letrozole alone. Based on the results of this study, letrozole plus CoQ10 is recommended to treat patients with iOAT syndrome who have high estradiol and low testosterone levels.

## Data Availability

Zenodo: Impact of coenzyme Q10 as an adjuvant therapy to letrozole on spermiogram results and sex hormone levels in Iraqi men with infertility; randomized open label comparative study. https://doi.org/10.5281/zenodo.8191739. ^38^ This project contains the following underlying and extended data:
- Raw spreadsheet data - Completed CONSORT checklist - Ethical approval of both committees Data are available under the terms of the Creative Commons Attribution 4.0 International license (CC-BY 4.0).
