# Supplementary material for: Impact of coenzyme Q10 as an adjuvant therapy to letrozole on spermiogram results and sex hormone levels in Iraqi men with infertility; randomized open label comparative study
Source: F1000Res. 2024 Jun 10;12:1093. Originally published 2023 Sep 1. [Version 3] doi: 10.12688/f1000research.131985.3 (PMC11217721; doi:10.12688/f1000research.131985.3)
Supplement: Peer review history

# Reviewer report: Reviewer response for version 3

**DOI:** 10.5256/f1000research.164830.r289156

**Published:** 2024-07-01

**Authors:** Qinghua Shi

**Affiliations:** 1 Division of Reproduction and Genetics, University of Science and Technology of China, Hefei, China

I have read the revised manuscript and found that the authors have responded to my concerns and made revisions according to my suggestions. I am generally satisfied with their revisions.

Is the work clearly and accurately presented and does it cite the current literature?

Partly

If applicable, is the statistical analysis and its interpretation appropriate?

Partly

Are all the source data underlying the results available to ensure full reproducibility?

Yes

Is the study design appropriate and is the work technically sound?

Partly

Are the conclusions drawn adequately supported by the results?

Partly

Are sufficient details of methods and analysis provided to allow replication by others?

Yes

Reviewer Expertise:

Spermatogenesis, reductive genetics, meiosis, infertility

I confirm that I have read this submission and believe that I have an appropriate level of expertise to confirm that it is of an acceptable scientific standard.

# Reviewer report: Reviewer response for version 3

**DOI:** 10.5256/f1000research.164830.r289157

**Published:** 2024-06-20

**Authors:** Neslihan Hekim

**Affiliations:** 1 Ondokuz Mayis University, Samsun, Turkey

The researchers have made the changes requested by the other reviewers and followed their suggestions. The study is suitable for indexing in this way.

Is the work clearly and accurately presented and does it cite the current literature?

Yes

If applicable, is the statistical analysis and its interpretation appropriate?

Partly

Are all the source data underlying the results available to ensure full reproducibility?

Yes

Is the study design appropriate and is the work technically sound?

Yes

Are the conclusions drawn adequately supported by the results?

Partly

Are sufficient details of methods and analysis provided to allow replication by others?

Partly

Reviewer Expertise:

Epigenetics, DNA damage, ROS, antioxidant capacities, sperm chromatin condensation in male infertility are some of my focuses about my studies.

I confirm that I have read this submission and believe that I have an appropriate level of expertise to confirm that it is of an acceptable scientific standard.

# Reviewer report: Reviewer response for version 2

**DOI:** 10.5256/f1000research.157556.r237937

**Published:** 2024-02-08

**Authors:** Neslihan Hekim

**Affiliations:** 1 Ondokuz Mayis University, Samsun, Turkey

Researchers aimed to evaluate the impacts of adding coenzyme Q10 to letrozole on the results of spermiogram and sex hormone tests in men diagnosed with idiopathic OAT. My suggestions and questions regarding the study are as follows:

1. It is not stated where participants received their OAT diagnosis.
2. Some cytogenetic and molecular genetic analysis results, which are commonly used in the evaluation of infertile patients, are not included in the exclusion criteria. Have participants' Y chromosome microdeletion or *CFTR* gene mutation analyzes been evaluated?
3. Why is the antioxidant usage period determined as three months?
4. How was Coenzyme Q10 concentration used by the participants determined?
5. In statistical analyses, the tests used to analyze the dependent variables should be added along with the analyzes of the independent variables.
6. It is known that smoking causes oxidative stress and it is also stated in the article. However, smoking was not analyzed in the study other than as demographic data. Researchers may have evaluated the parameters of smoker and non-smoker participants separately.
7. It should be mentioned in the discussion why semen volume increased in the group using only letrozole compared to the group using letrozole + coQ10.
8. The reason for the apparent effect of CoQ10 on sperm morphology is not mentioned in the discussion, but should be mentioned.

Is the work clearly and accurately presented and does it cite the current literature?

Yes

If applicable, is the statistical analysis and its interpretation appropriate?

Partly

Are all the source data underlying the results available to ensure full reproducibility?

Yes

Is the study design appropriate and is the work technically sound?

Yes

Are the conclusions drawn adequately supported by the results?

Partly

Are sufficient details of methods and analysis provided to allow replication by others?

Partly

Reviewer Expertise:

Epigenetics, DNA damage, ROS, antioxidant capacities, sperm chromatin condensation in male infertility are some of my focuses about my studies.

I confirm that I have read this submission and believe that I have an appropriate level of expertise to confirm that it is of an acceptable scientific standard, however I have significant reservations, as outlined above.

# Reviewer report: Reviewer response for version 2

**DOI:** 10.5256/f1000research.157556.r237934

**Published:** 2024-02-05

**Authors:** Qinghua Shi

**Affiliations:** 1 Division of Reproduction and Genetics, University of Science and Technology of China, Hefei, China

This manuscript presents the results of letrozole alone and with CoQ10 on semen parameters after one, two and three months-treatment on patients with iOAT, and achieved a promising conclusion. However, the number of patients receiving the treatment is small, research from more patients is needed to confirm its conclusion. Besides, some points need to be fixed:

 1. Recent and original research papers related to this topic should be cited.

 2. "LH stimulates Leydig cells to release testosterone to promote the production of sperm and virilization and exerts negative feedback to suppress ......FSH promotes Sertoli cells to support spermatogenesis to release inhibin B .....", Too many "to". The subject  before "exerts" is not clear.

 3. "......ecological factors (such as radiation and toxins) are.....", radiation and toxins are not and ecological factors.

 4. "The proportion of the control and intervention groups was 1:1 (balanced design)" is not correct.

 5. "Assessment of sperm volume/ ml, sperm concentration/ ml, total sperm count/ml, progressive sperm motility (%), non-progressive sperm motility (%), immotile sperm (%), normal sperm morphology (%)" should be: "Assessment of semen volume, sperm concentration, total sperm count, progressively motile sperm (%), non-progressively motile sperm (%), immotile sperm (%), morphologically normal sperm (%)".

 6. "Assessment of concentration of serum FSH, concentration of serum estradiol, and concentration of serum testosterone" should be "Assessment of concentration of serum FSH, estradiol, and testosterone."

 7. Table 2, the "/ml" in the first column must be deleted.

 8. In several Table legends, ""between (Time-wise values)" within each group" should be rephrased. "Different small case letters indicate significant difference." is not clear.

 9. Table 3, The name in the first column is inaccurate.

 10. Discussion, "the results indicated improvement in sperm motility, ....." ,  ".....levels showed an increase in FSH .....levels”, "The combined administration of letrozole and CoQ10 led to significant improvements of all spermiogram parameters," are not true. In "This combination appears to have a synergistic effect to obtain higher quality spermatogenesis", should be changed to "......to improve spermatogenesis".

 11. Conclusion, "The combined use of letrozole and CoQ10 was found to improve sperm parameters and patients showed a boosted improvement in all spermiogram parameters compared to that of patients administered letrozole alone.". The "all" should be removed. 

 12. Acknowledgments, "Dr Hayder A. Fawzi for its support and encouragement to", the "its" should be "his".

Is the work clearly and accurately presented and does it cite the current literature?

Partly

If applicable, is the statistical analysis and its interpretation appropriate?

Partly

Are all the source data underlying the results available to ensure full reproducibility?

Yes

Is the study design appropriate and is the work technically sound?

Partly

Are the conclusions drawn adequately supported by the results?

Partly

Are sufficient details of methods and analysis provided to allow replication by others?

Yes

Reviewer Expertise:

Spermatogenesis, reductive genetics, meiosis, infertility

I confirm that I have read this submission and believe that I have an appropriate level of expertise to state that I do not consider it to be of an acceptable scientific standard, for reasons outlined above.

# Reviewer report: Reviewer response for version 1

**DOI:** 10.5256/f1000research.144878.r204199

**Published:** 2023-09-13

**Authors:** Isarin Thanaboonyawat

**Affiliations:** 1 Infertility Unit, Department of Obstetrics and Gynecology, Faculty of Medicine, Siriraj Hospital, Mahidol University, Salaya, Nakhon Pathom, Thailand

This is a randomized, open-label comparative study of sperm quality assessment following combined treatment with coenzyme Q10 and letrozole versus letrozole alone. OAT is widely studied, however, the causes and treatment guidelines are not established. This study suggested a possible combination treatment, however, some key points need to be modified.

**Background:**

 The first two sentences are not relevant to the heading. It would be better to describe what is known, such as the effect of CoQ10 on sperm quality or male sex hormones; and what aspects need further exploration. Are there any similar studies published? It is better to summarize the results here.

**Introduction:**

 The content is well-written and easy to understand. Although the mechanisms of sperm protection by CoQ10 and letrozole are elucidated, a thorough review of published articles on the effects of CoQ10 and letrozole treatment on OAT is more important. The rationale for using both drugs in this study should be rigorously discussed. Did the author expect the synergistic function? The present introduction is better summarized to avoid a lengthy introduction.

 Cited references are usually those that provide evidence or information for the first time, rather than articles that reuse that information.

 Reference 1 needs to be the statement of WHO. Please check it carefully.

 References 2 and 3 are not the original articles that reported the prevalence of 15% or the prevalence of malefactors. Please cite the original article and authors who reported this information.

 Reference 6 is a minireview, not the original article that provided the information as cited. Please cite the original article and authors who reported this information.

 Reference 7 is a review of the significant aspects of irisin actions and its involvement in energy homeostasis and male reproduction. Please cite a more relevant article.

 “Idiopathic oligoasthenoteratozoospermia (iOAT) is associated with faulty spermatogenesis and is characterized by unusually low sperm count, motility, and a large number of dysmorphic spermatozoa in the ejaculate” Please define iOAT clearly with proper reference.

 Reference 17: “Oxidative stress (OS) and reactive oxygen species (ROS) are believed to damage the spermatozoa and account for 30 to 80% of infertility cases in men,” The original article needs to be cited.

**Methods:**

 “This registered with ClinicalTrials.gov (NCT05847257) on May 6, 2023.” The RCT was retrospectively registered.

 The authors are required to provide the details of methods to make the process reproducible.

 How to randomize the participants? Sealed envelope? How many sets? The proportion of the control and intervention groups is better stated. I suppose it was 1:1?

 How to determine the sample size? Did you estimate the sample size based on the semen concentration? What is the initial semen concentration and the expected concentration after treatment? If the authors used the semen concentration of Peivandi’s study, did you include the same kind of participants Peivandi included male patients with abnormal parameters of semen fluid based on WHO criteria, which were serum T/E2 ratio < 10 (testosterone mg/dl and estradiol pg/ml).

 For sample estimation: If the authors assessed multiple parameters, concentration, motility, and morphology, the largest sample size estimated from each parameter will be used. That means if the sample size needed based on concentrations is less than the sample size calculated based on motility, the sample size used for motility should be used.  Could you please provide the process of sample calculation?

 It is better to provide the definition of OAT? How many semen samples per patient were used? How much is the lag time between each sample collection?

 VIDAS® automated quantitative tests are used for plasma and serum. Semen has different components. Using the same instrument may produce different results compared to plasma. Could you please show the evidence to support to use of this instrument?

 How to measure the semen fluid volume, concentration, motility, etc. Please describe the procedures.

 The technique of semen preparation for hormonal measurement is crucial. Could you please state the procedures, such as any dilution or centrifugation? Were cryopreservation needed? How?

 For hormone measurements, please describe how the instrument was used, the settings used, and positive/negative control samples. How to interpret the results of the read. How about the CV of the measurement? What is the measurement range and sensitivity? This information will provide the validity of the study.

**Results:**

 CONSORT FLOW CHART

 Thirty-four and 40 patients were randomized in groups A and B, respectively. There were 5 and 2 patients lost in each group. Why were only 29 patients/group analyzed? Where were the other 9 patients in Group B?

 The authors presented the protocol analysis. If possible, ITT analysis will be more useful.

**Discussion:**

 Well written.

 Are there any explanations for the mechanisms of CoQ10 in improving sperm concentration?

Is the work clearly and accurately presented and does it cite the current literature?

Partly

If applicable, is the statistical analysis and its interpretation appropriate?

Partly

Are all the source data underlying the results available to ensure full reproducibility?

Partly

Is the study design appropriate and is the work technically sound?

Partly

Are the conclusions drawn adequately supported by the results?

Yes

Are sufficient details of methods and analysis provided to allow replication by others?

No

Reviewer Expertise:

Infertility, in vitro fertilization, fertility preservation, IVM, embryo culture, ovarian tissue cryopreservation, implantation, probiotics

I confirm that I have read this submission and believe that I have an appropriate level of expertise to confirm that it is of an acceptable scientific standard, however I have significant reservations, as outlined above.

# Response

**Published:** 2023-10-14

**Authors:** Hayder Fawzi

**Affiliations:** Pharmacy, Al-Mustafa University College, Baghdad, Iraq

Answer to reviewer 1

 Background:

 Q/ The first two sentences are not relevant to the heading. It would be better to describe what is known, such as the effect of CoQ10 on sperm quality or male sex hormones; and what aspects need further exploration. Are there any similar studies published? It is better to summarize the results here.

 A/ This article focuses first and most on infertility, so we started our article in defining the main issue based on well-known definition of WHO, and after we defined infertility, we introduced the intervention as you mentioned. Next the new background.

 “Infertility is common problem affecting many couples, still many interventions directed to treat infertility with varying outcomes. The aim of this clinical trial was to evaluate the impacts of adding coenzyme Q10 (CoQ10) to letrozole on the results of spermiogram and sex hormone tests in men diagnosed with idiopathic oligoasthenoteratozoospermia (iOAT) syndrome, which is a type of male defective spermatogenesis of unknown etiology.”

 Introduction:

 Q/ The content is well-written and easy to understand. Although the mechanisms of sperm protection by CoQ10 and letrozole are elucidated, a thorough review of published articles on the effects of CoQ10 and letrozole treatment on OAT is more important. The rationale for using both drugs in this study should be rigorously discussed. Did the author expect the synergistic function? The present introduction is better summarized to avoid a lengthy introduction.

 A/ During our initial proposal of the research, we kept in mind possible additive effect at least and at best synergic effect.

 Cited references are usually those that provide evidence or information for the first time, rather than articles that reuse that information.

 Q/ Reference 1 needs to be the statement of WHO. Please check it carefully.

 A/ We changed Ref 1 to more appropriately, as listed below:

 World Health Organisation. Infertility [Internet]. www.who.int. 2022. Available from: https://www.who.int/health-topics/infertility#tab=tab_1 [Accessed: February 2022]

 Q/ References 2 and 3 are not the original articles that reported the prevalence of 15% or the prevalence of malefactors. Please cite the original article and authors who reported this information.

 A/ Ref 2, changed to the following:

 “Sharlip ID, Jarow JP, Belker AM, Lipshultz LI *, et al.* Best practice policies for male infertility. Fertility and sterility. 2002;77(5):873-82.DOI:10.1016/S0015-0282(02)03105-9”

 Ref 3, changed to the following:

 Q/ Reference 6 is a minireview, not the original article that provided the information as cited. Please cite the original article and authors who reported this information.

 A/ Ref 6, changed to the following:

 Ma Y, He X, Qi K, Wang T *, et al.* Effects of environmental contaminants on fertility and reproductive health. Journal of Environmental Sciences. 2019;77:210-7.DOI: https://doi.org/10.1016/j.jes.2018.07.015

 Q/ Reference 7 is a review of the significant aspects of irisin actions and its involvement in energy homeostasis and male reproduction. Please cite a more relevant article.

 A/ Ref 7, changed to the following:

 “Kim HH, Schlegel PN. Endocrine manipulation in male infertility. The Urologic clinics of North America. 2008;35(2):303-18, x.DOI:10.1016/j.ucl.2008.01.003”

 Q/ “Idiopathic oligoasthenoteratozoospermia (iOAT) is associated with faulty spermatogenesis and is characterized by unusually low sperm count, motility, and a large number of dysmorphic spermatozoa in the ejaculate” Please define iOAT clearly with proper reference.

 A/ New definition:

 “Idiopathic male infertility is referred to the impairment of sperm parameters without clear male-associated cause, Although physical examination, endocrine, genetic, and biochemical laboratory results for these men are normal, semen analysis can show abnormal findings, they do not have a history of disorders that influence fertility” [1]

 Minhas S, Bettocchi C, Boeri L, Capogrosso P *, et al.* European Association of Urology Guidelines on Male Sexual and Reproductive Health: 2021 Update on Male Infertility. European urology. 2021;80(5):603-20.DOI:10.1016/j.eururo.2021.08.014

 Q/ Reference 17: “Oxidative stress (OS) and reactive oxygen species (ROS) are believed to damage the spermatozoa and account for 30 to 80% of infertility cases in men,” The original article needs to be cited.

 A/ Ref 17, changed to the following [2]:

 Showell MG, Mackenzie-Proctor R, Brown J, Yazdani A *, et al.* Antioxidants for male subfertility. Cochrane Database Syst Rev. 2014(12):Cd007411.DOI:10.1002/14651858.CD007411.pub3

 Methods

 Q/ “This registered with ClinicalTrials.gov (NCT05847257) on May 6, 2023.” The RCT was retrospectively registered.

 We gave our explanation inside the article, please refer to the designated section inside “ethical statement”, “ Due to the recent introduction of required clinical trials registration in Iraq, we registered the study retrospectively to assure its transparency.”

 Q/ The authors are required to provide the details of methods to make the process reproducible.

 We included all possible methodologies and we adhered to the CONSORT checklist, again if something specific is/are unclear please ask specifically and we will provide an answer.

 Q/ How to randomize the participants? Sealed envelope? How many sets? The proportion of the control and intervention groups is better stated. I suppose it was 1:1?

 We implemented a block randomization design in which we divided the participants into 14 blocks (sets) each with a block size of 6 patients stratified by treatment groups, see Link table.

 The proportion of the control and intervention groups was 1:1 (balanced design).

 Q/ How to determine the sample size? Did you estimate the sample size based on the semen concentration? What is the initial semen concentration and the expected concentration after treatment? If the authors used the semen concentration of Peivandi’s study, did you include the same kind of participants Peivandi included male patients with abnormal parameters of semen fluid based on WHO criteria, which were serum T/E2 ratio < 10 (testosterone mg/dl and estradiol pg/ml).

 We estimated the sample by A previous study by Peivandi *et al.*, showed that letrozole 2.5 mg increases sperm concentrations over three months and demonstrated an in improvement by 33% for sperm parameters (concentration after three months of therapy) so we assumed that the use of CoQ10 will increase this value to 55%. Using MedCalc (RRID:SCR_015044) software version 14.8.1, we arrived at 64 patients for both groups to confirm the null hypothesis (with type I error 5% and type II error of 5%), we increased the sample to 74 to account for maximum possible loss in patients being around 15% without affecting the accuracy of the results.

 Keep in mind that sample size is a method to estimate the sample size, within certain levels of certainty and best to relay on the trial outcomes.

 Q/ For sample estimation: If the authors assessed multiple parameters, concentration, motility, and morphology, the largest sample size estimated from each parameter will be used. That means if the sample size needed based on concentrations is less than the sample size calculated based on motility, the sample size used for motility should be used.  Could you please provide the process of sample calculation?

 Yes, the largest sample size for sperm concentrations was used. And attached is the output of the MedCal program. See linked figure.

 Q/ It is better to provide the definition of OAT? How many semen samples per patient were used? How much is the lag time between each sample collection?

 We included a definition of OAT in the introduction

 The semen sample was collected four times per patient (once at baseline, after 1 month, after 2 months, and after 3 months) each time we took a single semen sample at each visit. All this information is stated in the article already!

 Q/ VIDAS® automated quantitative tests are used for plasma and serum. Semen has different components. Using the same instrument may produce different results compared to plasma. Could you please show the evidence to support to use of this instrument?

 VIDAS is used for assessing serum markers which we mentioned in the “variable measurement” section, it can not measure the semen parameters we measured which include: “sperm volume/ ml, sperm concentration/ ml, total sperm count/ml, progressive sperm motility (%), non-progressive sperm motility (%), immotile sperm (%), normal sperm morphology (%).” We measured using the WHO methods for assessing semen parameters, we added the details inside the new version of the article under the new section “semen fluid analysis”.

 The samples were collected via masturbation after abstinence of 3 to 4 days directly into a dry, clean disposable wide-mouth plastic container in a private room close to the laboratory in the institute. Immediately after that the samples were carried to laboratory of semen examination then put in an incubator at 37ºC for 30 minutes. After complete liquefaction, the semen analysis was done by macroscopic and microscopic examination according to standard criteria of WHO (2021) Ref: World Health Organization. WHO laboratory manual for the examination and processing of human semen Sixth Edition. 2021.

 Q/ How to measure the semen fluid volume, concentration, motility, etc. Please describe the procedures.

 We add a new section that explains seman measurement.

 Q/ The technique of semen preparation for hormonal measurement is crucial. Could you please state the procedures, such as any dilution or centrifugation? Were cryopreservation needed? How?

 Regarding these points, we only used VIDIS to assess the hormonal levels, we used the manual methods for assessing the semen parameters and we separated these details inside the article.

 Q/ For hormone measurements, please describe how the instrument was used, the settings used, and positive/negative control samples. How to interpret the results of the read. How about the CV of the measurement? What is the measurement range and sensitivity? This information will provide the validity of the study.

 FSH [VIDAS®FSH, bioMerieux SA, France Cat no.:30 407-01, (Measurement range: 0.1–110 mIU/mL, Detection limit: ≤0.1, Intra-assay CV: ≤5%, Inter-assay CV: ≤6%)”

 estradiol [VIDAS®E2II, bioMerieux SA, France Cat no.: 30 431, “Measurement range: 9 – 3000 pg/mL, Detection limit: 9 pg/mL, Intra-assay CV: ≤7.5%, and Inter-assay CV: ≤9.5%)”]

 testosterone [VIDAS® Testosterone II, bioMerieux SA, France Cat no.:414320], “Measurement range: 0.1 – 13 ng/mL, Detection limit: 0.1 ng/mL, Intra-assay CV: ≤10%, and Inter-assay CV: ≤4.5%)”]

 VIDAS is an automated device that gives you the interpretation directly, please refer to the manufacturer's website ( https://www.biomerieux-nordic.com/product/vidasr-fertility-panel ).

 Results:

 Q/ CONSORT FLOW CHART

 Thirty-four and 40 patients were randomized in groups A and B, respectively. There were 5 and 2 patients lost in each group. Why were only 29 patients/group analyzed? Where were the other 9 patients in Group B?

 The authors presented the protocol analysis. If possible, ITT analysis will be more useful.

 Thank you for your note we had an error in filling the flow chart and we updated it in the article, see linked figure.

 Discussion:

 Well written.

 Q/ Are there any explanations for the mechanisms of CoQ10 in improving sperm concentration?

 Co Q10 has a powerful antioxidant scavenging free radical and involved in production of mitochondrial energy which is important for maintaining spermatozoa's energy, also protects its membrane from damage by peroxidation of lipid.

 Ref: Salvio G, Cutini M, Ciarloni A, Giovannini L, Perrone M, Balercia G. Coenzyme Q10 and Male Infertility: A Systematic Review. Antioxidants (Basel, Switzerland) 2021;10(

 References

 1.       Minhas S, Bettocchi C, Boeri L, Capogrosso P *, et al.* European Association of Urology Guidelines on Male Sexual and Reproductive Health: 2021 Update on Male Infertility. European urology. 2021;80(5):603-20.DOI:10.1016/j.eururo.2021.08.014

 2.       Showell MG, Mackenzie-Proctor R, Brown J, Yazdani A *, et al.* Antioxidants for male subfertility. Cochrane Database Syst Rev. 2014(12):Cd007411.DOI:10.1002/14651858.CD007411.pub3
